# Co-Expression of Mutant Tau and α-Synuclein in Neurons Promotes Tau Phosphorylation, Neuronal Loss, and Neuroinflammation in Mouse Brain

**DOI:** 10.1007/s12035-025-05248-y

**Published:** 2025-07-25

**Authors:** Yuki Yamamoto, Toshiki Kubota, Daisuke Noguchi, Takaomi C. Saido, Toshio Ohshima

**Affiliations:** 1 Department of Life Science and Medical Bioscience, Waseda University, 2-2 Wakamatsu-cho, Shinjuku-ku, Tokyo, 162-8480 Japan; 2 Laboratory for Proteolytic Neuroscience, RIKEN Center for Brain Science, 2-1 Hirosawa, Wako-shi, Saitama, 351-0198 Japan

**Keywords:** Aggregation, Accumulation, Tauopathy

## Abstract

Intracellular aggregation and accumulation of protein is a hallmark of neurodegenerative diseases. Tauopathy, which is caused by aggregated tau accumulation, is a group of neurodegenerative diseases, including frontotemporal dementia (FTD), Pick disease, and Alzheimer’s disease. Similarly, synucleinopathy, which is caused by aggregated α-synuclein (α-syn) accumulation, includes Parkinson’s disease and dementia with Lewy body (DLB). The interaction between tau and α-syn has been attracting attention because of similarities in symptoms and the co-existence of tau and α-syn in neural cells. Previous studies revealed that tau and α-syn promote their aggregation with each other. Additionally, other studies showed that α-syn promotes tau spreading in the mouse brain. In the present study, we investigated the relationship between tau and α-syn and the effects of their co-existence in neuronal cells on mouse pathology by double transgenic strategy. Consequently, we found increased phosphorylated tau, a declined number of neurons in the CA1 region, and increased astrocyte and microglia in the hippocampi in double transgenic mice at 8 months old. In mice that co-express tau and α-syn, locomotive activity increased and cognitive function decreased in behavioral test. These results suggest the co-existence of tau andα-syn in neurons that promote neuronal loss and impaired cognitive function in neurodegenerative conditions.

**Supplementary Information:**

The online version contains supplementary material available at 10.1007/s12035-025-05248-y.

## Introduction

Aggregation and accumulation of the protein are the hallmark of neurodegenerative disease [1]. Tauopathy and synucleinopathy are the groups within the neurodegenerative disease. Tauopathy is caused by aggregation and accumulation of tau, a member of the microtubule associated protein family, including Alzheimer’s disease and Pick disease [2]. Synucleinopathy is caused by aggregation of α-synuclein(α-syn), a soluble protein that locates to the nerve terminal, including Parkinson’s disease (PD) and dementia with Lewy body [3, 4].

Colocalization of tau and α-syn are reported in some studies and suggest the relationship between tauopathy and synucleinopathy [5, 6]. Previous studies show that tau and α-syn have the cross-talk and may progress the disease. In in vivo studies, the C-terminal of α-syn interacts with tau [7]. Additionally, α-syn promotes phosphorylation of tau via protein kinase (PKA) and GSK3β [7–10]. Some in vitro studies found that tau and α-syn promote aggregation and fibrillation in each other [11, 12]. Additionally, a previous study found that α-syn promotes tau spreading in the brain by injecting protein fibril into the mice brain [13]. Conversely, whether tau promotes α-syn spreading in the brain remains unclear because there are papers showing opposing results [14, 15].

Previously, P301S mutation on tau has been reported as a familial mutation of frontotemporal dementia with parkinsonism [16]. P301S mutation promotes filament formation more than WT tau, and tau accumulates with age in P301S tau Tg mouse brain [17, 18]. A53T mutation on α-syn has been reported as a familial mutation of PD. A53T α-syn turned to aggregate formation more than WT α-syn in vitro [19]. In the mice model, phosphorylated α-syn was observed at the hippocampus in 5-month-old A53T Tg mice [20].

Microglia and astrocytes are important regulators of inflammatory responses in the central nervous system [21]. Neuroinflammation is a protective mechanism for the brain and has beneficial effects; however, long-term inflammatory responses have been shown to be detrimental [22–24]. Tau and α-syn activate microglia and astrocytes that release inflammatory cytokines. Tau activates microglia that lead to the overproduction of pro-inflammatory cytokines via the p38 MAPK pathway [25]. Moreover, α-syn oligomers induce microglia into an activated phenotype and promote the release of inflammatory cytokines (TNF-α, IL-1β), leading to CNS inflammation and neuronal death [26, 27]. Tau fibrils convert astrocytes to an activated phenotype that releases pro-inflammatory cytokines and chemokines [28]. Moreover, astrocytes with accumulated α-syn release pro-inflammatory cytokines (TNF-α, IL-1, IL-6) and chemokines (CXCL1 and CX3CL1) and promote neuroinflammation [29, 30].

Previous studies showed the interaction of α-syn and tau co-aggregation and co-accumulation by mating of transgenic mice. Clinton et al. crossed 3xTg (APP/PS1/tau) andα-syn Tg and demonstrated advanced pathologies in tauopathy and synucleinopathy [31]. In another study, α-syn and tau are overexpressed in oligodendrocyte and showed co-aggregation and co-accumulation in oligodendrocyte [12]. Presently, we co-expressed human mutantα-syn (A53T) and tau (P301S) in neurons by crossing α-syn A53T Tg [18] and PS19 Tg [32] mice to examine the effect of the co-existence of tau and α-syn in neuronal cells. In double transgenic mice, we observed the advancement of tauopathy, neuronal loss in the hippocampus, and cognitive impairment.

## Materials and Methods

### Animals

All animal experiments were conducted in accordance with approved protocols from Waseda University’s Institutional Animal Care and Use Committee. The study utilized female wild-type (WT) mice alongside three transgenic models: P301S Tau transgenic mice (Tau Tg) expressing human microtubule-associated protein tau (MAPT) 1N4R tau with the P301S mutation under the mouse prion promoter (MoPrP) [18], and A53T α-synuclein transgenic mice (α-syn Tg) expressing the A53T mutant variant under the same promoter (stock 006823, Jackson Labs; Bar Harbor, ME, USA) [32]. Heterozygous α-syn Tg line was maintained by crossbreeding with WT mice. P301S Tau Tg; A53T α-syn heterozygous mice (Double Tg mice) were obtained by mating Tau Tg female and α-syn Tg male mice and genotyped as described [18, 32]. To investigate disease progression, the same cohort of mice was analyzed longitudinally at 5 and 8 months of age.

### Histological Analysis

Mice were fully anesthetized using a combination of three types of mixed anesthetic agents—medetomidine (0.75 mg/kg, Dorbene®; Kyoritsu, Tokyo, Japan), midazolam (5 mg/kg, Sandoz K. K. Tokyo, Japan), and butorphanol (4 mg/kg Vetorphale®; Meiji Seika Pharma Co., Ltd., Tokyo, Japan)—and perfused with phosphate-buffered saline (PBS) and 4% paraformaldehyde (PFA) in PBS. The brain samples were fixed in a 4% PFA solution overnight (O/N) at 4 °C. Brain samples were rinsed with PBS and sequentially dehydrated in 10%, 20%, and 30% sucrose solutions (prepared in PBS). After dehydration, the samples were embedded with optimal cutting temperature (O.C.T.) compound (Tissue-Tek®). The frozen sections were cut into 14 µm thick coronal sections using a cryostat (Microm HM500M: Leica). The sections were stuck on a Matsunami Advanced Surface (MAS)-coated glass slide (S9441; Matsunami) and stored at −20 °C.

For immunostaining, we used the following primary antibodies: anti-NeuN (rabbit polyclonal, GTX133127, GeneTex), anti-phosphorylated-α-synuclein (p-α-syn) Ser129 (rabbit, ab51253, Abcam), anti-phosphorylated-tau AT8 clone targeting pS202/pT205 (mouse monoclonal, MN1020, Invitrogen), anti-Iba1 (rabbit, 019-19741, Wako), and anti-glial fibrillary acidic protein (GFAP) (mouse, 131-17719, Invitrogen). The brain tissue sections were rinsed in PBS for 30 min and then permeabilized in PBS containing 0.1% Triton X-100 and 3% horse serum for 10 min. For AT8 immunostaining, tissue sections underwent antigen retrieval via heating in citrate buffer at 85 °C. Sections were blocked with 3% horse serum in 0.01% Triton X-100/PBS (PBSTr) for 1 h, followed by incubation with primary antibody in the same blocking solution at 4 °C O/N. Sections were rinsed in PBSTr for 10 min thrice and incubated with secondary antibody and Hoechst in 3% horse serum at room temperature for 1 h. After incubation, the sections were rinsed in PBSTr for 10 min thrice again and coated with Fluoromount aqueous mounting medium (Sigma-Aldrich). The images were obtained using a confocal laser microscope FV3000 (Olympus). Immunostaining intensity for p-α-syn Ser129 in selected hippocampal CA1 regions was quantified using ImageJ software (National Institutes of Health) [20]. For quantification of immunostaining, all images were captured using identical exposure times within each antibody staining set to ensure comparable intensity measurements across sections. To quantify positively stained cells, we established intensity thresholds based on the background staining levels specific to each antibody. Cells were counted as positive only when immunostaining co-localized with Hoechst nuclear staining. Counts were performed across four representative sections per mouse.

### Behavioral Analysis

#### Open Field Test

Open-field test equipment and systems (O'Hara and Co., Tokyo, Japan) were used to perform the open field test, as described [33]. Mice were placed in the center of a box (50 × 50 × 25 cm), and their behavior was recorded for 10 min using a camera installed above the box. The box was divided into 25 squares (10 × 10 cm); the middle 9 squares were considered the “center region” (Sup Fig. 1a). Total distance traveled and average speed of the mice running in the box were measured. Additionally, time spent in the center region was measured.

**Fig. 1:**
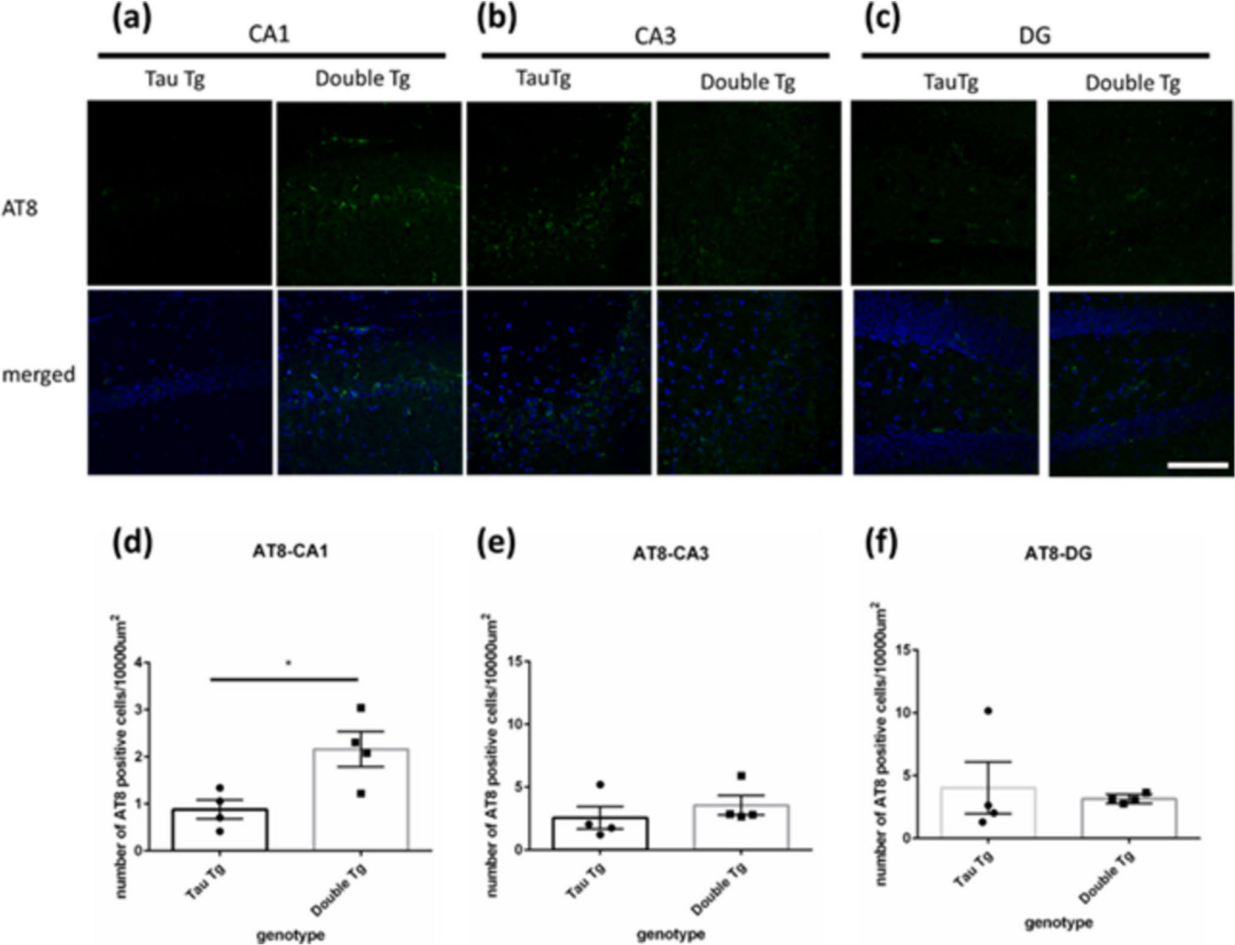
The number of AT8 positive cells in hippocampus of 8-month-old mice. **a–c** Images of immunostaining with anti-AT8 antibody (green, p-Tau are stained) and Hochest (blue, nuclei are stained) in CA1 (**a**), CA3 (**b**), and DG (**c**) region of the hippocampus in 8-month-old Tau and double Tg female mice. Scale bar = 100 µm. **d–f** Counting result of the number of AT8 positive cells in the CA1 (**d**), CA3 (**e**), and DG (**f**) region per 10000 µm^2^. Data are shown with mean ± SEM. Welch’s *t*-test. *p<0.05, number of mice=4, 4

#### Contextual Fear Conditioning Test

Fear conditioning test equipment and systems (O’Hara and Co., Tokyo, Japan) were used to perform the contextual fear conditioning test, as described [20]. An acrylic chamber was used to provide the mice with foot shocks and measure their behavior. Metallic grates were placed on the chamber’s floor to provide electric shocks to the mice. On day 1, the mice were placed in the chamber and provided with three foot shocks (2.0 mV for 2 s) during a 5-min measurement. On day 2, the mice were placed in the same chamber but were not subjected to foot shock during the 5-min measurement (Fig. 6a). All measurements were recorded using a camera installed above the chamber. The measurements were automatically analyzed using a fear conditioning system. Freezing behavior was defined as the absence of movement or movement less than 300 pixels for ≥ 2 s. Freezing time was calculated as freezing percentage.

### Statistical Analysis

All data are shown as mean ± SEM. All datasets of behavioral analysis were evaluated for normality using Shapiro–Wilk tests. Non-normally distributed datasets were analyzed using Kruskal–Wallis non-parametric tests. In other cases, statistical analysis was performed using an unpaired one-way ANOVA with Tukey’s multiple comparison test or Welch’s *t*-test (confidence interval 95%). The statistical methods used for each analysis are shown in figure legends.

## Results

### Accumulation of Phosphorylated Tau in the CA1 Region Was Increased by Co-Expressing Tau P301 and α-Syn A53T in 8-Month-Old Mice

To observe the effect of α-syn and tau double mutations on the amount of tau aggregation in the hippocampus, we performed immunostaining using an anti-phosphorylated tau antibody (AT8, pS202, and pT205 tau) in 8-month-old females. Consequently, cells with p-tau aggregation were found in the CA1, CA3, and dentate gyrus (DG) regions of Tau Tg and double Tg mice (Fig. 1a–c). We counted the cells where the AT8 positive signals overlapped with the nuclei or where the signals were close to the nucleus as AT8 positive cells. We found that AT8 positive cells in the CA1 region of double Tg mice were significantly increased compared to that in Tau Tg mice (Fig. 1a, d). However, there was no significant difference in the CA3 and DG regions between Tau Tg and double Tg mice. In the hippocampi of double Tg mice, the accumulation of phosphorylated proteins increased compared to that in Tau Tg mice.

### Comparison of Phosphorylated α-Syn in the Hippocampus Induced by Co-Expression of Tau P301 and α-Syn A53T in 8-Month-Old Mice

**Fig. 2:**
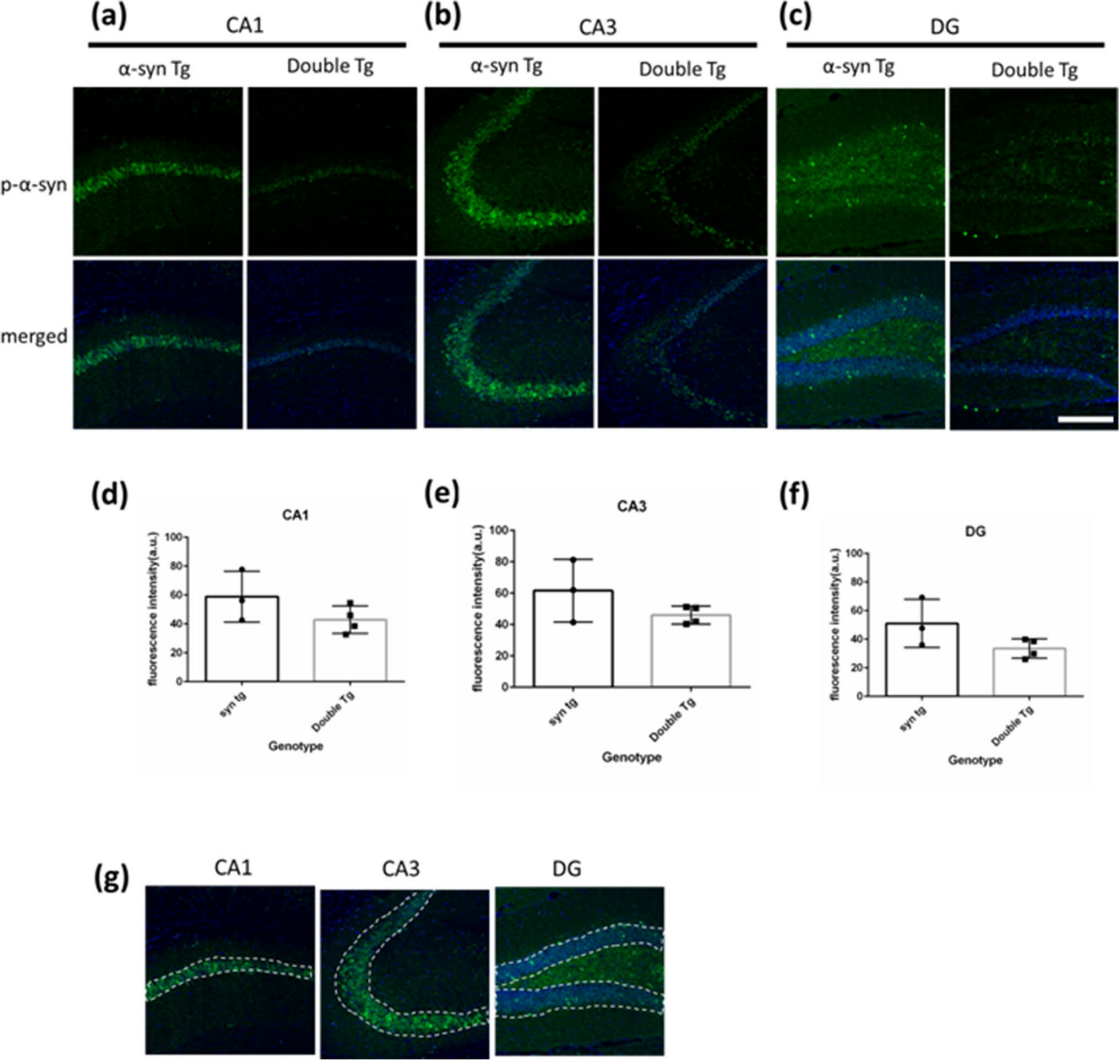
The p-α-synuclein positive intensity in hippocampus of 8-month-old mice. **a–c** Images of immunostaining with anti-phospho α-synuclein (phospho S129) antibody (green, phospho S129 α-syn are stained) and Hochest (blue, nuclei are stained) in CA1, CA1, and DG region of the hippocampus in 8-month-old p-α-syn and double Tg mice. Scale bar = 200 µm. **d–f** Quantification result of the mean gray value about p-α-syn (phosphor S129) positive intensity in CA1 region (**d**), CA3 region (**e**), and DG region (**f**). Data are shown with mean ± SEM. Welch’s *t*-test. Number of mice=3, 4. **g** The area for quantification

To assess the impact of α-syn and tau double mutation on hippocampal α-syn accumulation, we conducted immunostaining with an anti-phosphorylated α-syn antibody (pS129) in 8-month-old female mice, as previously described [20]. Consequently, p-α-syn accumulation was found in cells of the hippocampal CA1, CA3, and DG regions in α-syn and double Tg mice (Fig. 2). Given the difficulty in quantifying all p-α-syn-positive cells, analysis of the intensity of p-α-syn immunostaining served as an indicator of p-α-syn accumulation levels within the identified regions (Fig. 2a–c). No significant changes were observed across the hippocampal CA1, CA3, and DG regions between α-syn and double Tg mice (Fig. 2d–f).

### Number of Microglia and Astrocyte in Hippocampus Increased by Co-Expressing Tau P301 and α-Syn A53T in 8-Month-Old Mice

**Fig. 3:**
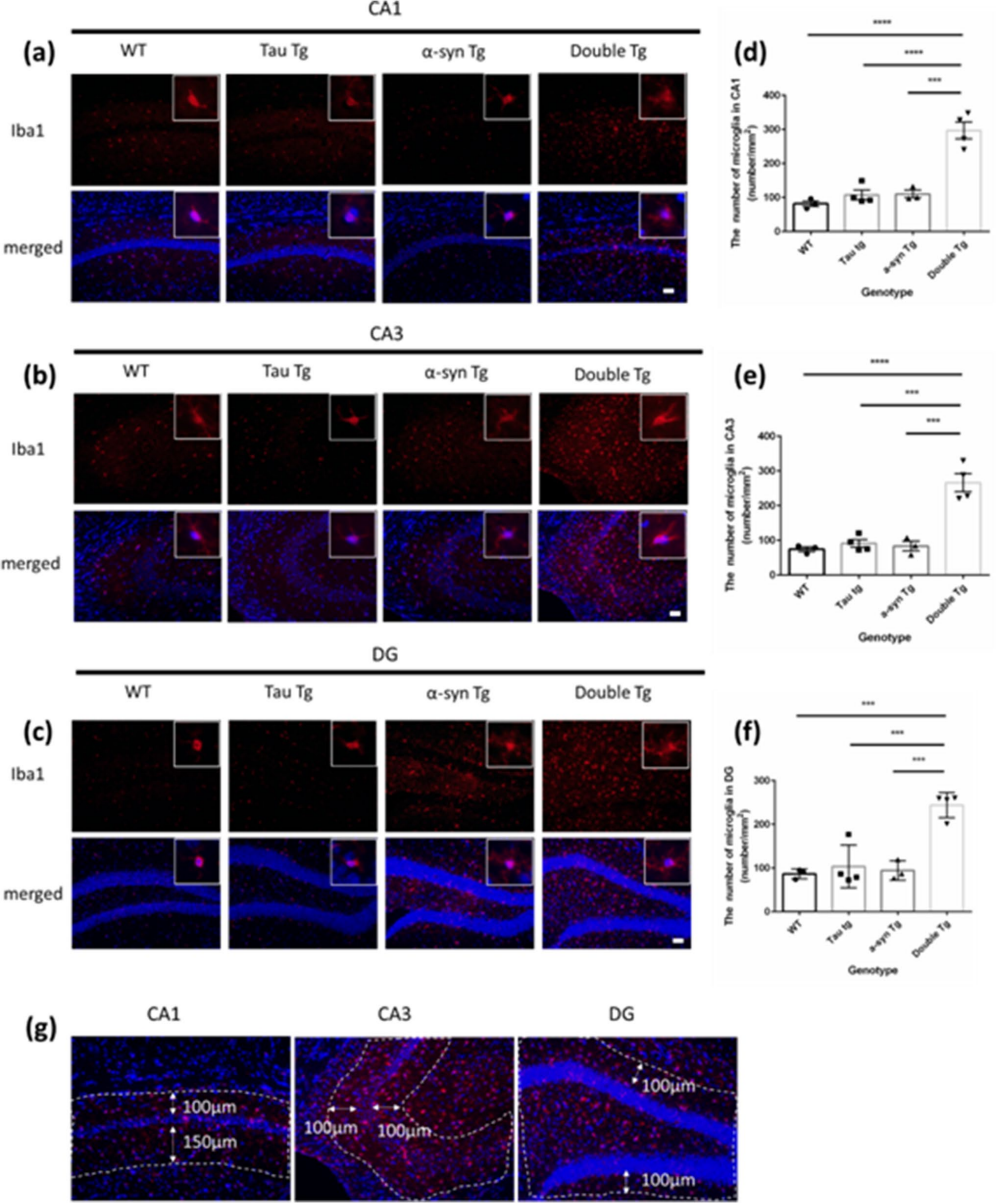
The number of Iba1 positive cells in hippocampus of 8-month-old mice. **a–c** Images of immunostaining with anti-Iba1 antibody (red, microglia are stained) and Hochest (blue, nuclei are stained) in CA1 (**a**), CA3 (**b**), and DG (**c**) region of the hippocampus in 8-month-old WT, Tau Tg, p-α-syn Tg, and double Tg mice. Scale bar = 50 μm. **d–f** Counting result of the number of microglia (Iba1 positive cells) in the CA1 (**d**), CA3 (**e**), and DG (**f**) region per mm^2^ in female mice. Data are shown with mean ± SEM. One-way ANOVA with Tukey’s multiple comparison. ***p<0.001, ****p<0.0001. Number of mice=3, 4, 3, 4. **g** The area for quantification

To observe the extent of microglial inflammation in α-syn and tau double mutation against other genotypes, we performed immunostaining using anti-Iba1 antibody on 8-month-old female mice. We quantified Iba1-positive cells when signals overlapped with the nucleus or surrounded the nucleus in the identification area (Fig. 3a–c, g) as Iba1-positive cells. We found that the number of microglia in the CA1, CA3, and DG regions of the double Tg mice showed a significant increase compared to that in WT, Tau Tg, and α-syn Tg mice (Fig. 3d–f). However, no significant differences were observed among WT, Tau Tg, and α-syn Tg groups (Fig. 3d–f).

**Fig. 4:**
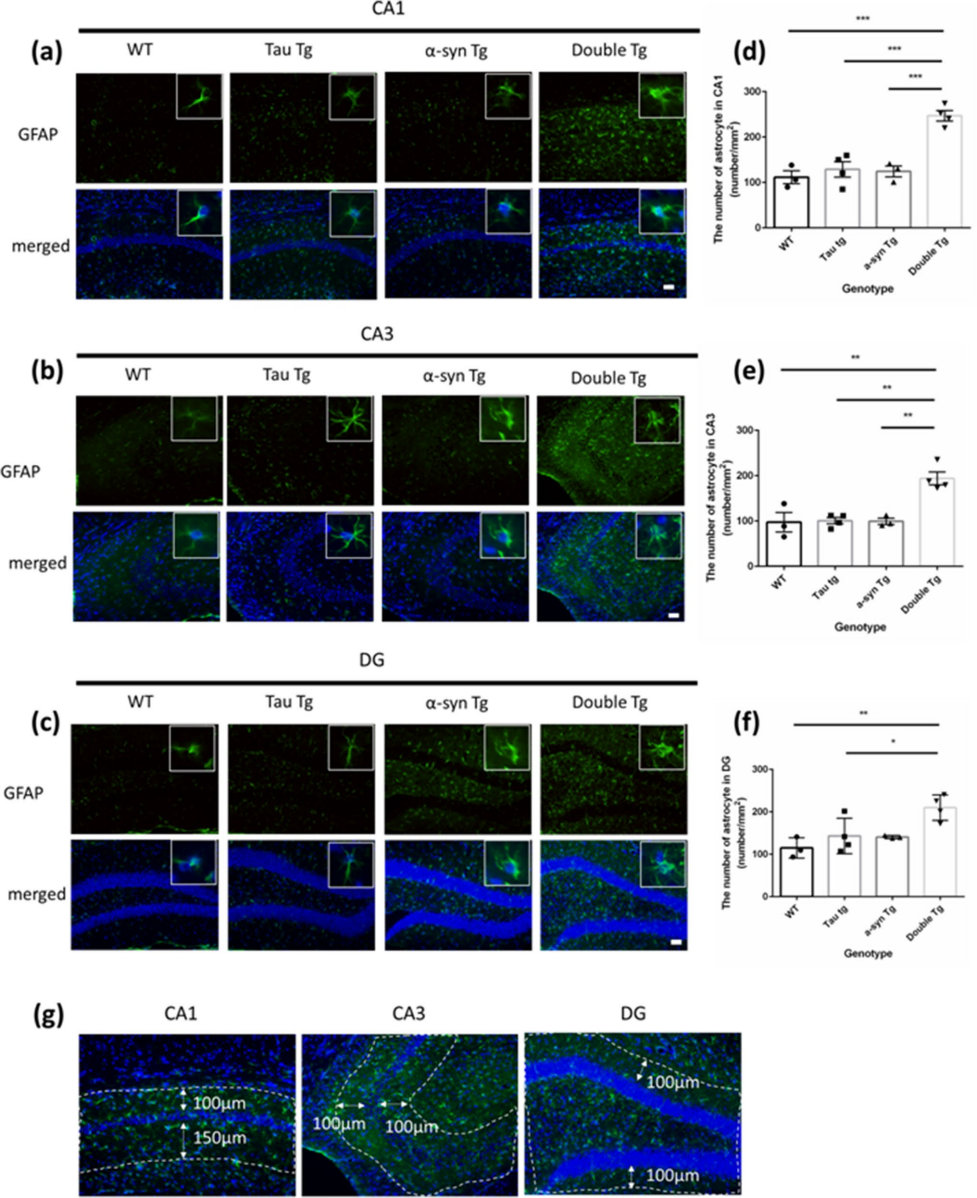
The number of GFAP positive cells in hippocampus of 8-month-old mice. **a–c** Images of immunostaining with anti-GFAP antibody (green, astrocyte are stained) and Hochest (blue, nuclei are stained) in CA1 (**a**), CA3 (**b**), and DG (**c**) region of the hippocampus in 8-month-old WT, Tau Tg, p-α-syn Tg, and double Tg mice. Scale bar = 50 μm. **d–f** Counting result of the number of astrocyte (GFAP positive cells) in the CA1 (**d**), CA3 (**e**), and DG (**f**) region per mm^2^ in female mice. Data are shown with mean ± SEM. One-way ANOVA with Tukey’s multiple comparison. *p<0.05, **p<0.01, ***p<0.001. Number of mice=3, 4, 3, 4. **g** The area for quantification

To further assess the extent of astrocytic activation in α-syn and tau double mutations compared to other genotypes, we conducted GFAP immunostaining in 8-month-old female mice. We quantified GFAP-positive cells when signals overlapped with the nucleus or surrounded the nucleus in the identification area (Fig. 4g). Astrocyte numbers in the CA1 and CA3 regions in double Tg mice showed significant increase compared to that in WT, Tau Tg, and α-syn Tg mice (Fig. 4d, e).

In the DG region, the number of astrocytes was significantly higher in double Tg mice than in WT and Tau Tg groups. There was no significant difference between double Tg and α-syn Tg mice, but the number of double Tg mice tended to increase compared to that of α-syn Tg mice. No relevant changes were found in WT, Tau Tg, and α-syn Tg groups. In summary, astrocyte and microglia counts were higher in double Tg mice compared to that in the other genetic groups.

### Number of Neurons Decreased in the CA1 Region of Tau P301 and α-Syn A53T Co-Expressing 8-Month-Old Female Mice

**Fig. 5:**
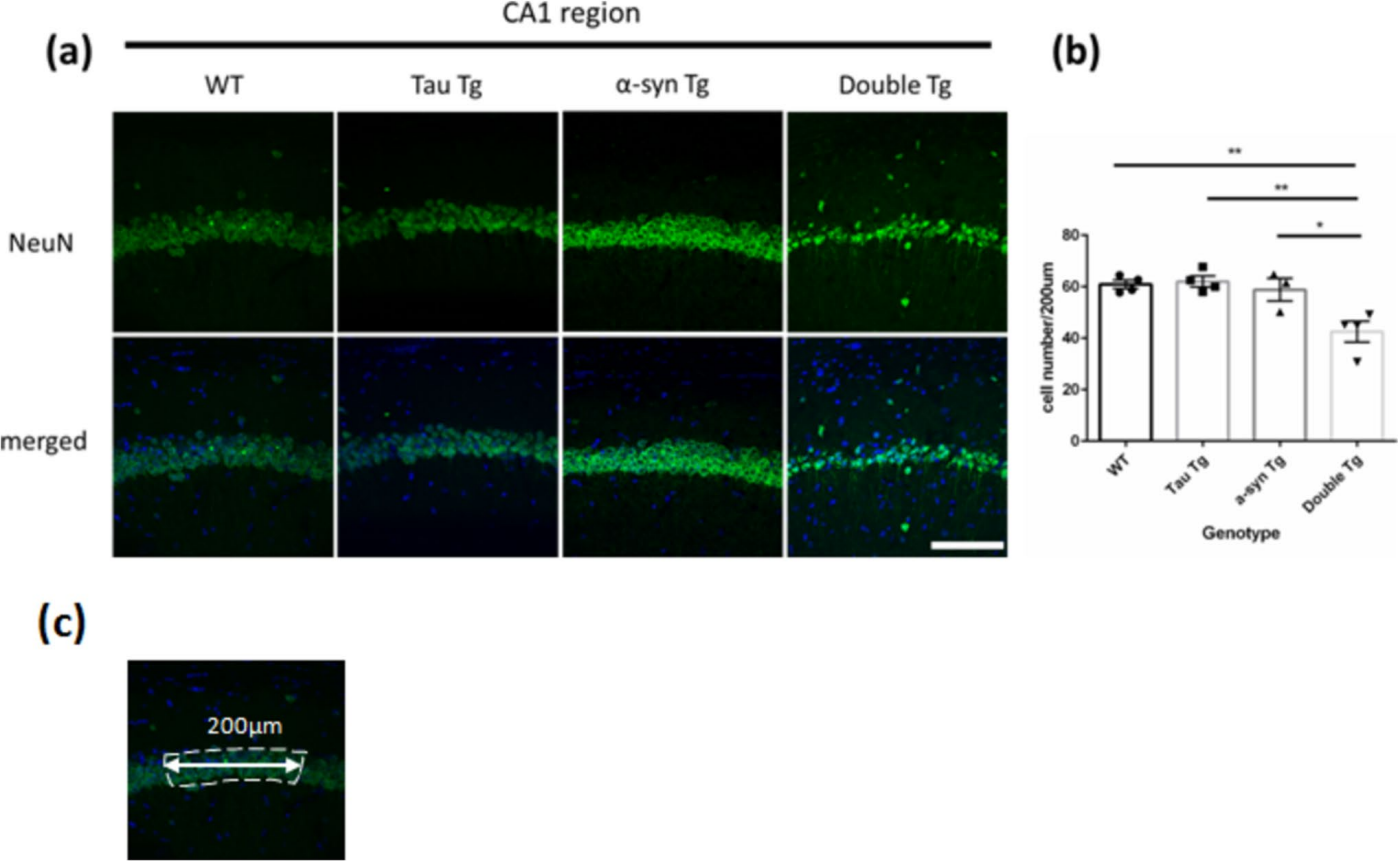
The number of NeuN positive cells in CA1 region of hippocampus of 8-month-old mice. **a** Images of immunostaining with anti-NeuN antibody (green, neuron are stained) and Hochest (blue, nuclei are stained) in the CA1 region of the hippocampus in 8-month-old WT, Tau Tg, p-α-syn Tg, and double Tg mice. Scale bar = 1.0 µm. **b** Counting result of the number of neuron (NeuN positive cells) in the CA1 region per 200 µm. Data are shown with mean ± SEM. One-way ANOVA with Tukey’s multiple comparison. *p<0.05, **p<0.01. Number of mice=4, 4, 3, 4. **c** The area for quantification

To observe the impact of α-syn and tau double mutation on neuronal density in the hippocampal CA1 region, NeuN immunostaining was performed in 8-month-old mice (Fig. 5a). NeuN-positive cells were quantified when signals overlapped with the nucleus or surrounded the nucleus. The number of neurons in the CA1 of double Tg mice was significantly lower than other genotype groups in 8-month-old mice (Fig. 5b).

### Co-Expression of Tau P301 and α-Syn A53T Increased Locomotive Activity in Female Mice

To evaluate the behavioral effects of tau and α-syn double mutations, open field testing was conducted in 5- and 8-month-old mice. Five-month-old female double Tg mice exhibited significantly greater total distance traveled and higher moving speed compared to that of both WT and Tau Tg mice. Similarly, 8-month-old female double Tg mice demonstrated significantly increased locomotion metrics than did WT controls (Sup Fig. 1b, c). α-syn Tg mice exhibited significantly greater total distance traveled and higher moving speed than did both WT and Tau Tg mice (Sup Fig. 1b, c). No significant differences in locomotion metrics were observed between α-syn Tg and double Tg in 8-month-old females.

### Co-Expression of Tau P301 and α-Syn A53T Promoted Impairment of Cognitive Function in 8-Month-Old Female Mice

**Fig. 6:**
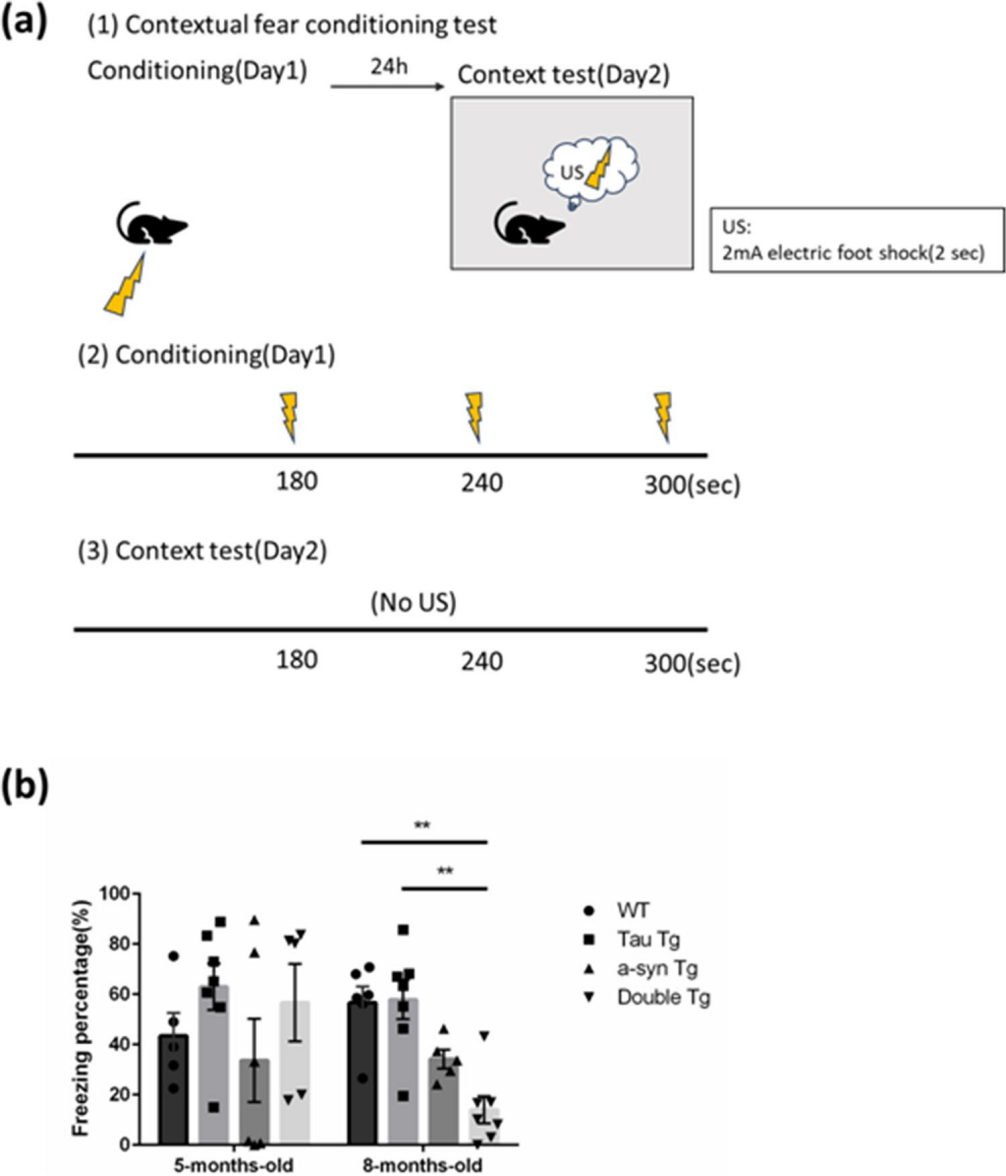
The difference between genotype on cognitive function impairment in the fear conditioning test. **a** Overview of the contextual fear conditioning test. **b** The total freezing percent of day 2 of 5- and 8-month-old mice. Data are shown with mean ± SEM. Kruskal-Wallis test. **p<0.01. 5-month-old; number of mice=5, 7, 6, 5; 8-month-old; number of mice = 6, 7, 5, 7

The fear conditioning test did not show any differences between 5- and 6-month-old male Tau Tg and WT mice [34]. Moreover, compared to WT controls, 5-month-old female α-syn Tg mice exhibited significantly reduced freezing percentages [20]. To investigate the cognitive effects of tau and α-syn double mutations, contextual fear conditioning tests were performed in 5- and 8-month-old mice. Five-month-old female mice showed no significant genotype-dependent differences (Fig. 6b). However, compared to WT and Tau Tg mice, 8-month-old female double Tg mice displayed significantly lower freezing percentages (Fig. 6b). Although no significant difference emerged between α-syn Tg and double Tg females, the double Tg group showed a tendency toward reduced freezing percentage compared to that by α-syn Tg mice (Fig. 6b). These results indicate an age-dependent cognitive decline in female double Tg mice by 8 months of age.

## Discussion

To study the interactions between tauopathy and synucleinopathy, patient-derived tau and α-syn fibrils were intracerebrally inoculated into mouse brains [15], successfully demonstrating enhanced fibril propagation. To study the neuronal effects of co-existing tau and α-syn pathologies, we adopted a double transgenic method by crossing α-syn and tau transgenic lines.

This study showed greater p-tau accumulation in the hippocampal CA1 region of double Tg mice compared to that in Tau Tg mice, as revealed by AT8 immunostaining (targeting p-S202/p-T205 tau epitopes; Fig. 1a–c). Co-expression of α-syn and tau in mice increased p-tau accumulation, consistent with previous studies showing that tau increases in the presence of α-syn. As mentioned, α-syn promotes tau phosphorylation via protein kinase A (PKA) and glycogen synthase kinase 3β (GSK3β) activation [7–10]. In α-syn/tau co-expressing mice, p-tau aggregation reflects this increased kinase-mediated tau phosphorylation cascade.

While p-α-syn accumulated in the hippocampus of double Tg mice, the level of accumulation did not differ from that in α-syn Tg mice. This contrasts with in vitro findings demonstrating tau-mediated enhancement of α-syn aggregation [11, 12]. Our p-α-syn immunostaining analysis detected no exacerbated α-syn pathology in double Tg mice relative to α-syn Tg groups (Fig. 2). However, we cannot rule out that these negative results reflect methodological limitations, such as a potential “saturation” effect in the current staining and quantification approach. Additional studies using alternative detection methods are required to fully evaluate α-syn aggregates accumulation.

Previous studies of APP/PS1/Tau/α-syn transgenic mice demonstrated advanced synucleinopathy [31]. Bassil et al. reported amyloid-beta-mediated promotion of α-syn spreading and aggregation in dystrophic neurites [35]. Our double transgenic α-synuclein/tau model did not find advanced synucleinopathy. This suggests that amyloid pathology in 3xTg mice may contribute to the advanced synucleinopathy observed in the multiple transgenic model proposed by Clinton et al. [31].

The present study revealed increased astrocyte and microglia numbers in the hippocampus of double Tg mice, revealed via GFAP and Iba1 immunostaining (Figs. 3 and 4). These findings indicate enhanced glial activation in double Tg mice relative to WT controls, concomitant with elevated p-tau accumulation. This aligns with established reports that tau and α-syn aggregates activate glial cells [25–30]. P-tau increased accumulation, resulting from the co-expression of tau and α-syn in double Tg mice, likely contributed to this glial activation. Furthermore, double Tg mice exhibited a reduced neuronal density in the CA1 region (Fig. 5). Since neuronal cell death triggers astrocytes and microglia recruitment and facilitates clearance of cellular debris [36], the hippocampal CA1 region’s neuronal loss in double Tg mice may have led to an increase in astrocytes and microglia.

This study revealed a decrease in neuronal density in the hippocampal CA1 region of double Tg mice through NeuN immunostaining (Fig. 5a). Prior research demonstrates that activated astrocytes and microglia can directly damage neurons and induce neuronal death [37–42], while p-tau exhibits reduced microtubule binding capacity and is cytotoxic and neurotoxic [35, 36]. In Tau and α-syn Tg mice, neuroinflammation typically precedes neuronal loss and hippocampal volume reduction [18, 20]. It has been suggested that increased p-tau-positive cells in the CA1 region of double Tg mice and astrocytes and microglial cells are related to neuronal decrease. However, a direct relationship between neuroinflammation and cell death requires further investigation. While we focused on CA1 neuronal loss, other hippocampal regions, including the CA3 and DG subregions, remains unexamined. Although brain atrophy emerges by 9 months of age in Tau Tg mice [18], and other Tau overexpression models demonstrate clear neuron-loss associations [43, 44], our study did not fully elucidate how α-syn and p-tau interact to drive neuronal loss. Given previous studies have reported a link between tauopathy and neuroinflammation [45–47], a more comprehensive analysis was needed in our model.

The behavioral tests performed in this study revealed early onset hyperactivity in 5-month-old female double Tg mice (Sup Fig. 1) and later cognitive decline at 8 months (Fig. 6). This was revealed using behavioral, open-field, and contextual fear conditioning tests. While previous reports identified motor abnormalities in male Tau Tg mice preceding tau accumulation [48, 49], we observed no such hyperactivity in female Tau Tg mice at 5 or 8 months of age. Female PS19 mice exhibit slower disease progression than do males [50]. Sex-associated differences remain a topic for future studies. The hyperactivity in 5-month-old double Tg females reflects synergistic effects of combined tau and α-syn pathology, besides α-syn’s individual hyperactivity contribution [51]. The fear conditioning test showed no differences between 5- and 6-month-old male Tau Tg and WT mice [42]. Given the importance of the dorsal hippocampus’ role in contextual learning [52], CA1 commissural pathway potentiation and increased excitation of Schaffer collateral-CA1 dendrite synapses after a fear conditioning test [53, 54] suggest the involvement of CA1 regions in contextual learning. Particularly, we suggest that the reduced freezing percentages in 8-month-old double Tg mice may be largely related to reduced hippocampal function and CA1 neuronal loss observed in our study.

Several limitations warrant mention. Histological analysis was not performed at 5 months of age in the behaviorally tested cohort because of the involvement of a longitudinal 8-month analysis, which is necessary to investigate the relationship between pathological progression and abnormalities in behavioral analysis. The present study was conducted using hemizygotes for P301S tau and/or A53T α-syn Tg mice. Future studies should investigate homozygous models to further explore these interactions. Based on our previous findings demonstrating hippocampal p-α-syn accumulation and contextual learning deficits in 5-month-old hemizygous A53T Tg mice [20], we selected hemizygous P301S and/or A53T Tg models for this study, anticipating more severe phenotypic manifestations in double Tg mice.

Overall, the co-expression of tau and α-syn may increase p-tau aggregation, leading to worsening neuroinflammation, associated neurodegeneration, increased motor abnormalities, and decreased cognitive function. Further studies are warranted to identify the potentially therapeutic elements in α-syn and tau-associated neurodegenerative diseases.

## Supplementary Information

Below is the link to the electronic supplementary material.

Supplementary file1 (DOCX 144 KB)

## Data Availability

No datasets were generated or analysed during the current study.

## Abbreviations

α-syn: α-Synuclein
DLB: Dementia with Lewy body (DLB)
FTD: Frontotemporal dementia
O.C.T.: Optimal cutting temperature
O/N: Overnight
p-α-syn: Phosphorylatedα-syn
PBS: Phosphate-buffered saline
PFA: Paraformaldehyde
PKA: Protein kinase A
Tg: Transgenic
WT: Wild-type
