# Supplementary material for: Co-Expression of Mutant Tau and α-Synuclein in Neurons Promotes Tau Phosphorylation, Neuronal Loss, and Neuroinflammation in Mouse Brain
Source: Mol Neurobiol. 2025 Jul 25;62(12):15832–43. doi: 10.1007/s12035-025-05248-y (PMC12559100; doi:10.1007/s12035-025-05248-y)
Supplement: Supplementary file 1 — Supplementary file1 (DOCX 144 KB) [file 12035_2025_5248_MOESM1_ESM.docx]

**Supplemental Information**

**Co-expression of mutant tau and α-synuclein in neurons promotes tau phosphorylation, neuronal loss and neuroinflammation in mouse brain**

Yuki Yamamoto^1^, Toshiki Kubota^1^, Daisuke Noguchi^1^, Takaomi C Saido^2^ and Toshio Ohshima^1,3^


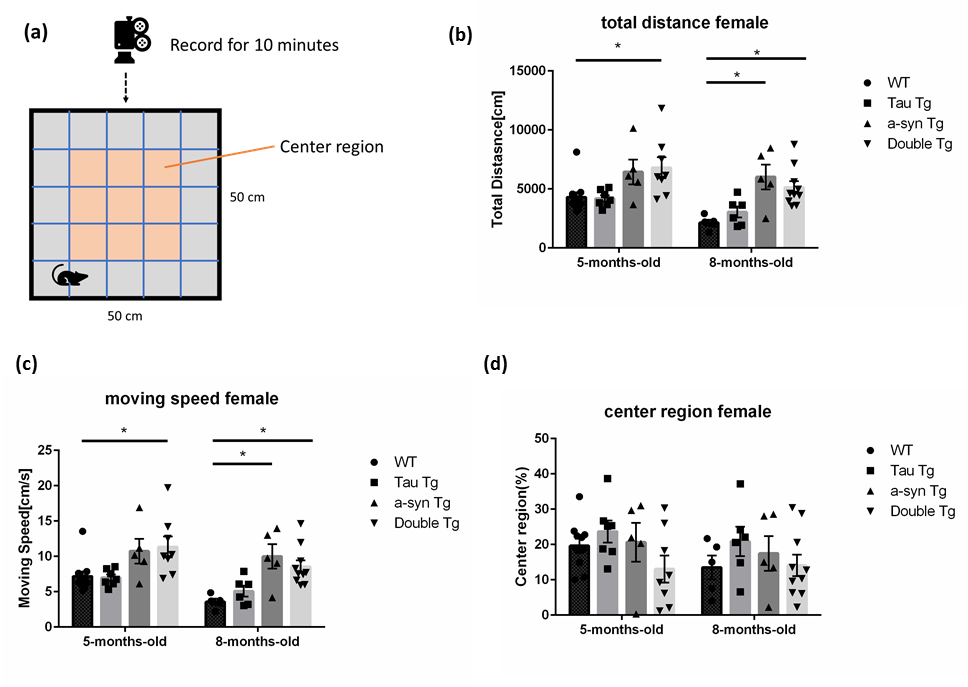


**Supplemental Fig.1. The difference between genotype on amount of activity in open field test**

(a) Overview of the open field test. (b) The total distance that the mice run in the field in 10 min of 5- and 8-month-old mice. (c) The average moving speed that the mice run in the field in 10 min of 5- and 8-months-old mice. (d) The rate of time that mice stayed in center region in the field in 10 min of 5- and 8-months-old mice. Data are shown with mean±SEM. Kruskal-Wallis test. *p<0.05. 5-month-old; number of mice=11, 7, 5, 8; 8-month-old; number of mice=5, 6, 5, 1.
